# Genome Sequence of *Bacillus amyloliquefaciens* GB03, an Active Ingredient of the First Commercial Biological Control Product

**DOI:** 10.1128/genomeA.01092-14

**Published:** 2014-10-30

**Authors:** Soo-Keun Choi, Haeyoung Jeong, Joseph W. Kloepper, Choong-Min Ryu

**Affiliations:** a Super-Bacteria Research Center, Korea Research Institute of Bioscience and Biotechnology (KRIBB), Daejeon, Republic of Korea; b Korean Bioinformation Center, KRIBB, Daejeon, Republic of Korea; c Department of Entomology and Plant Pathology, Auburn University, Auburn, Alabama, USA

## Abstract

*Bacillus amyloliquefaciens* GB03 has been used as a representative commercialized strain of the bacilli for biological control against a broad spectrum of plant pathogens and as a bio-fertilizer to promote growth and yield of field crops for more than two decades. Herein, we present the genome sequence and a brief analysis of strain GB03.

## GENOME ANNOUNCEMENT

*Bacillus* spp. offer many benefits over Gram-negative bacteria for use as biological control agents, including a long shelf life under starvation or unfavorable conditions due to their ability to form endospores (1). Accordingly, several commercial products, such as Quantum, Kodiak, BioYield, Epic, Rhizo-Plus, Serenade, Subtilex, and System 3, that originated from *Bacillus* spp. are available in the U.S. and in other countries (1). *Bacillus amyloliquefaciens* strain GB03, originally described as *Bacillus subtilis* strain A13, was originally isolated from the healthy foliage of a Douglas fir in Australia (2). In an early study, the strain demonstrated plant growth promotion capacity in the field (3). A comprehensive mechanism study indicated that *B. amyloliquefaciens* GB03 was an efficient root colonizer that directly competes with potential root pathogens for nutrients and space at the surface of roots and produces antifungal agents such as iturins (4). *B. amyloliquefaciens* GB03 also reduces disease by inducing the plant’s innate immunity, a process referred to as induced systemic resistance (4). *Bacillus amyloliquefaciens* GB03, the active ingredient of Kodiak and Quantum, is a representative strain of the bacilli developed as a biological control agent against soilborne fungal pathogens (4).

Genome sequencing of *Bacillus amyloliquefaciens* GB03 was carried out at the National Instrumentation Center for Environmental Management (Seoul, Korea) using an Illumina Genome Analyzer IIx. A total of 27,492,038 reads (2.17 Gb, 101 nucleotides) were produced from paired-end sequencing of a genomic library with an average insert size of 393 bp. Sequence Read Archive (SRA) data are available under the accession no. SRS505354. *De novo* assembly using CLC Genomics Workbench version 4.8, after quality trimming and filtering (about 564-fold coverage after pretreatment of the reads), resulted in 37 contigs with a total length of 3,849,547 bp. The *N*_50_ and maximum contig length were 387,471 bp and 784,095 bp, respectively. Automatic genome annotation, performed using the RAST server (5), predicted 87 RNA genes and 3,892 coding sequences, 48% of which were categorized into subsystems. The G+C content was 46.6%. While the RAST analysis result suggested *Bacillus subtilis* subsp. *subtilis* 168 was the closest neighbor to the strain GB03, average nucleotide identity (ANI) analysis by the JSpecies program (6) revealed that *B. amyloliquefaciens* FZB42 and *B. amyloliquefaciens* Y2 are most similar to GB03 (>98.3% ANI values) among the completely sequenced *Bacillus* genomes.

Genome analysis revealed that nonribosomal peptide synthetase and polyketide synthase gene clusters for the production of surfactin, bacillomycinD, fengycin, bacillibactin, bacilysin, macrolactin, bacillaene, and difficidin are well conserved between strains GB03 and FZB42. However, the *nrsABCDEF* gene cluster of FZB42 was not found in the GB03 genome. Although the amino acid sequence of ComP has 68% identity between GB03 and FZB42, other genes related to bacterial traits for plant interactions such as root colonization, swarming, biofilm formation, biofertilization, and production of phytohormones like indole acetic acid and 2,3-butanediol are well conserved (7). In conclusion, the genome information of the GB03 strain will be helpful for strain improvement and for understanding the interaction between microorganisms and plants.

### Nucleotide sequence accession numbers.

This whole-genome shotgun project has been deposited at DDBJ/EMBL/GenBank under the accession no. AYTJ00000000. The version described in this paper is version AYTJ01000000.
